# Consumption of Alcopops During Brain Maturation Period: Higher Impact of Fructose Than Ethanol on Brain Metabolism

**DOI:** 10.3389/fnut.2018.00033

**Published:** 2018-05-08

**Authors:** Dounia El Hamrani, Henri Gin, Jean-Louis Gallis, Anne-Karine Bouzier-Sore, Marie-Christine Beauvieux

**Affiliations:** ^1^UMR5536 Centre de Resonance Magnetique des Systemes Biologiques (CRMSB), Centre National de la Recherche Scientifique (CNRS), Université de Bordeaux, LabEx TRAIL, Bordeaux, France; ^2^Service de Nutrition et Diabétologie, Hôpital Haut-Lévêque, Pessac, France

**Keywords:** high fructose, moderate ethanol, alcopops, designer drinks, ^13^C NMR, brain metabolism, astrocytes, rat

## Abstract

Alcopops are flavored alcoholic beverages sweetened by sodas, known to contain fructose. These drinks have the goal of democratizing alcohol among young consumers (12–17 years old) and in the past few years have been considered as fashionable amongst teenagers. Adolescence, however, is a key period for brain maturation, occurring in the prefrontal cortex and limbic system until 21 years old. Therefore, this drinking behavior has become a public health concern. Despite the extensive literature concerning the respective impacts of either fructose or ethanol on brain, the effects following joint consumption of these substrates remains unknown. Our objective was to study the early brain modifications induced by a combined diet of high fructose (20%) and moderate amount of alcohol in young rats by ^13^C Nuclear Magnetic Resonance (NMR) spectroscopy. Wistar rats had isocaloric pair-fed diets containing fructose (HF, 20%), ethanol (Et, 0.5 g/day/kg) or both substrates at the same time (HFEt). After 6 weeks of diet, the rats were infused with ^13^C-glucose and brain perchloric acid extracts were analyzed by NMR spectroscopy (^1^H and ^13^C). Surprisingly, the most important modifications of brain metabolism were observed under fructose diet. Alterations, observed after only 6 weeks of diet, show that the brain is vulnerable at the metabolic level to fructose consumption during late-adolescence throughout adulthood in rats. The main result was an increase in oxidative metabolism compared to glycolysis, which may impact lactate levels in the brain and may, at least partially, explain memory impairment in teenagers consuming alcopops.

## Introduction

Regular consumption of “alcopops,” also called “designer drinks” or “ready to drink” has increased among young people in Western societies (1), even if it varies within different countries and if no reliable survey can be found on teenagers (2). These flavored alcoholic beverages are sweetened by soft drinks containing fructose, which insidiously facilitates alcohol consumption among young consumers (3). Flavor, alcohol-strength, portability and low cost also influence young consumers (12–17 years old) toward this category of beverages (4), which can lead to episodic heavy drinking and related injuries (3). Considering that brain maturation in the prefrontal cortex and limbic system continues until 21 years of age, this drinking behavior is becoming a prevalent public health concern (5).

In Western diets, fructose is one of the most widely added sugars and its dietary intake is mostly through sucrose (50:50 molar mixture of fructose and glucose) and high fructose corn syrup (HFCS; 42, 55, or 90% of fructose). Compared to a natural consumption of 16–24 g daily with fruits and honey, “industrial” fructose consumption can reach 80 g/day which equates to 17–20% of the daily caloric intake (6). The NHANES (National Health and Nutrition Examination Survey) has reported that soft drinks account for 40% of the total daily intake of added sugar (7). An increasing literature has linked a high fructose (HF) diet with deleterious metabolic effects such as obesity, insulin resistance, hyperlipidemia and non-alcoholic hepatic steatosis [for review see (8–10)].

In addition, mounting evidence suggests that high fructose consumption can induce cerebral abnormalities. Indeed, HF diet alters spatial memory of rats exposed either during adulthood (60% fructose for 19 weeks) or a period considered as adolescence (11% sucrose or HFCS55 during 30 days) (11, 12). Moreover, 55% of rats fed on a HF diet during adolescence showed anxiety-like and depression-like behaviors with an elevated basal corticosterone concentration (13). In addition to the behavioral features, a HF diet induces alterations in the hippocampus (structure implicated in spatial memory), reduces neurogenesis associated with increased apoptosis (14), raises neuroinflammatory markers (interleukins 1β and 6) (12) and cerebral insulin resistance (15–17). Moreover, it has been hypothesized that regular dietary intake of fructose can play a role in the pathophysiology of chronic neurodegenerative diseases such as dementia (18, 19).

Alcohol drinking behaviors can be broadly divided in three categories: acute ethanol consumption (“binge drinking”; > 100 g ethanol or five “standard drinks” within 2 h), heavily chronic consumption of ethanol (alcoholism; > 80 g daily), and light to moderate consumption (<40 g from daily to weekly frequency) (20). There is very extensive literature concerning moderate alcohol consumption and its cerebral effects have been largely studied. It has been documented that ethanol interacts with neurotransmitter systems (dopamine, serotonin, GABA, glutamate) as well as second messenger systems (21).

Early moderate ethanol exposure in rats during the critical developmental period of adolescence has been reported to reduce hippocampal neurogenesis (22) and to lead to disturbances in the nucleus accumbens (structure of mesolimbic reward system) by increasing dopamine levels (23) and neuronal activation markers (24). Similarly, chronic moderate consumption in adult rodents induced alterations in dopaminergic neurotransmission in the nucleus accumbens (25), GABAergic disinhibition in the dorsolateral striatum (26), altered hippocampal glutamate basal levels (27), and reduced neurogenesis in the hippocampus contributing to changes in structural plasticity (28).

Recently, it has been proposed that fructose consumption has similar toxic effects to ethanol consumption, particularly in terms of hepatic metabolism (29). Indeed, fructose and ethanol are nonessential, insulin-independent, energetic substrates leading to hepatic steatosis. According to this hypothesis, it has been demonstrated that a mixed diet of high fructose and ethanol exacerbates hepatic steatosis accompanied by glucose metabolism impairment and dyslipidemia (30). It has also been shown that high fructose diet (60%) may potentiate the effects of chronic alcohol consumption by enhancing the hepatic inflammatory response (31).

Despite the extensive literature concerning the respective impacts of either fructose or ethanol on the brain, the effects following combined consumption of these substrates remains unknown. To our knowledge, it is the first time that the joint consumption of fructose and ethanol at dietary relevant concentrations has been studied for its impact on young rat brain metabolism. Our objective was to study the early brain modifications induced by a diet of high fructose (20%) combined or not with a moderate amount of alcohol in young rats by ^13^C-Nuclear Magnetic Resonance (NMR) spectroscopy, which is a powerful tool to follow the fate of enriched glucose (the main brain substrate) through cerebral metabolic pathways.

## Materials and methods

### Animals and diets

Male Wistar rats aged 7 weeks and weighing 230 g upon arrival were purchased from Janvier Labs (Le Genest Saint Isle, France). Rats were housed in cages inside a room with controlled parameters; temperature 21–23°C, hygrometry 30% and a 12 h light/dark system. The diets were started 4 days after their arrival and maintained for 6 weeks.

The protocol included four experimental groups (*n* = 8 for each group): (i) control diet (CT) with a standard chow (A04 SAFE, Augy, France); (ii) high fructose diet (HF) with 20% fructose (Sigma) added to the drinking water; (iii) ethanol group (Et) with a moderate dose of 0.5 g/kg of body weight (BW) per day (anhydrous absolute ethanol; Carlo Erba Reagents) added to the drinking water; (iv) a diet combining fructose and ethanol (HFEt) concentrations of groups (ii) and (iii) in drinking water.

The solid chow composition was similar in all groups and contained 60% carbohydrates, 16% proteins, 4% cellulose, 3% lipids, and 12% water. Diets were calculated to be isocaloric and pair fed among the groups based on Table 1. Diets were 10 g standard chow/100 g BW/day and 10 mL drinks/100 g BW/day. Rat weight and food consumption were measured every 48 h. This study was performed only on males to avoid the confounding factors linked to sex differences in puberty and hormone cycles. All procedures have been approved by the Institutional Animal Research Ethics Review Board of Bordeaux University (n° 5012029A) and followed the guidelines of the French governmental agency.

**Table 1:** Caloric value for each substrate.

Standard chow diet	2.9 kcal/g
High fructose 20%	0.8 kcal/mL
Ethanol	0.036 kcal/mL
High fructose 20% + ethanol	0.836 kcal/mL

### Infusion of [1-^13^C]glucose

At the end of the feeding period, the rats were anesthetized with an intraperitoneal injection of chloral hydrate (8%, 1 mL/200 g BW; Sigma-Aldrich, USA) and placed on a heated pad (37°C). [1-^13^C]D-glucose (750 mM, Cambridge Isotope Laboratories, Inc.) was infused into the tail vein over a total period of 60 min, following a previous 20 steps, time-decreasing exponential rate protocol going from 15–1.23 ml/h during the first 25 min after which the rate was kept unchanged for the last 35 min. This infusion protocol was designed to reach constant [1-^13^C]D-glucose concentrations in the blood and the total amount of glucose administered was 2.19 mmol/h per 200 g BW.

### Blood and brain samples

Prior to the infusion, 500 μL of blood was collected from the tail vein with heparinized syringes, centrifuged (1,500 g, 10 min) and plasma stored at −80°C. At the end of the infusion period and while the rats were still anesthetized, the abdomen was opened sufficiently to collect 2 mL of blood from the inferior vena cava into heparinized syringes. Blood samples were immediately centrifuged (1,500 g, 10 min) and plasma was kept at −80°C until it was analyzed. After blood sampling, rats were rapidly euthanized by focused brain scientific microwaves (5 kW, 1 s; Sacron8000, Sairem, Neyron, France), which completely prevents post-mortem metabolic modifications in the brain. The skull was cut opened with a micro-circular saw, the brain rapidly removed and dipped into liquid nitrogen. Brains were kept at −80°C until the metabolites were extracted.

### Perchloric acid extraction

Before extraction, each brain was weighed to normalize the data. Water soluble metabolites of the whole brain were extracted into perchloric acid (0.9 M) as previously described (32) with the following modifications. After homogenization, the suspension was centrifuged at 3,000 g for 10 min (4°C). The supernatant was neutralized at pH 7.20 with a 9 M KOH solution and centrifuged to eliminate perchlorate salts. Samples were then lyophilized. Prior to NMR spectroscopy, lyophilysates were dissolved in 400 μL of D_2_O and 10 μL of ethylene glycol (1 mmol/L in D_2_O) was added as an external reference.

### High resolution at the magic angle spinning (HRMAS) NMR spectroscopy

HRMAS NMR spectroscopy was performed on an 11.7T spectrometer (DPX 500MHz, Bruker Biospin, Wissembourg, France). For each brain perchloric acid extract, 50 μL was placed in a 4 mm-diameter rotor and analyzed at room temperature.

^1^H-NMR spectra were acquired with a 90° pulse angle (adjusted for each sample), relaxation time of 8 s, relaxation delay of 8 s, and spectrum width of 10 parts per million (ppm), acquisition time of 3.28 s, 128 scans and 32 K memory size. Homonuclear presaturation was used to suppress the water signal.

The specific enrichment (% ^13^C) of glucose carbon 1 (Glc C1 SEnr) was determined from the ratio of the integral of Glc satellite peaks resulting from heteronuclear spin-coupling to the sum of the integrals of satellite and central peaks.

^1^H-decoupled ^13^C-NMR spectra were acquired with a 90° pulse angle, relaxation time of 20 s, 200 ppm spectrum width, 1.31 s acquisition time, ~5,500 scans, and 64 K memory size. From these ^13^C spectra, relative enrichment of glutamate (Glu) C2/C3 and glutamine (Gln) C2/C3 were calculated.

^1^H-observed/^13^C-edited (POCE) NMR spectra were acquired under ^13^C decoupling as previously described (32). The method included a first scan consisting of a standard spin-echo acquisition in which ^1^H linked to all carbons (^12^C and ^13^C) were detected, and a second scan corresponding to a ^13^C inversion pulse, in which only ^1^H coupled to ^13^C were visible. Brain metabolite ^13^C enrichments were determined from the ratio of the integral of a resonance on the edited ^13^C-^1^H spectrum to its integral in the standard spin-echo spectrum.

### Plasma metabolites

Plasma samples were collected at the end of the ^13^C-glucose infusion and analyzed using a clinical chemistry analyzer (Olympus AU2700, BCO, Villepinte, France) with kits for glucose (Glc), proteins, albumin, total cholesterol, HDL-cholesterol, triglycerides (TG) and free fatty acids (FFA). These assays were performed in the medical laboratories of Bordeaux Hospital (Haut-Levêque, France). Insulin levels were assessed by ELISA (Diasorin, Antony, France) at the Institut de Chimie et Biologie des Membranes et des Nano-objets (Pessac, France).

### Data analysis

Values are mean ± SEM. Statistical analyses were performed using Prism 5 (GraphPad, La Jolla, CA, USA). Two way-ANOVA analyses followed by a *post-hoc* Bonferroni test were used to compare body weight as a function of diet and time. Statistical comparisons between diets for plasma and brain metabolites were performed using non-parametric one-way ANOVA (Kruskal–Wallis analysis followed by a *post-hoc* test of Dunn). A *p* < 0.05 was considered statistically significant.

## Results

### Body mass and food intake

**Figure 1:**
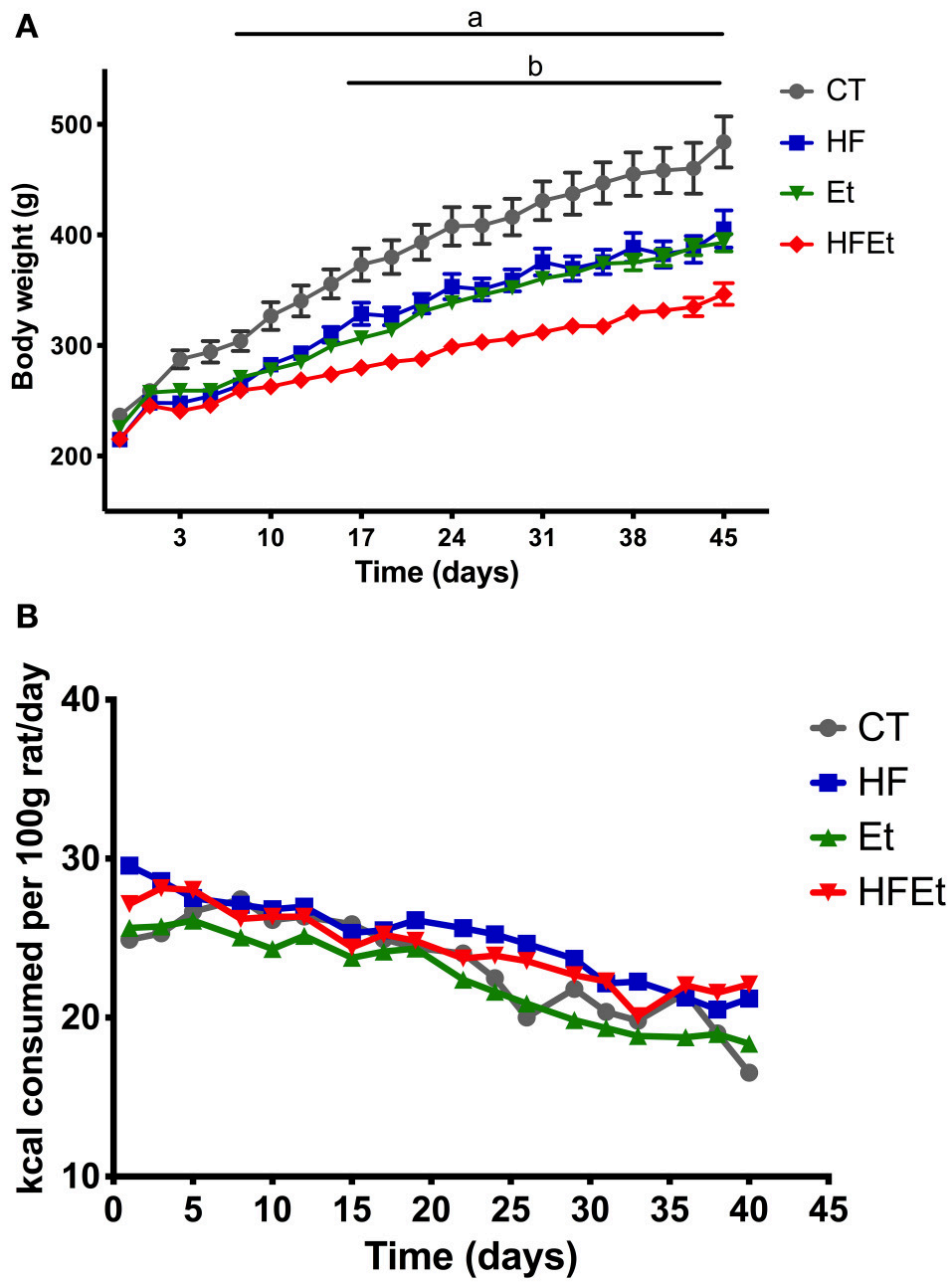
Body weight **(A)** and food intake **(B)** of rats fed with control diet (CT), high fructose diet (HF), ethanol (Et), or a mix (HFEt) during 6 weeks. Values are mean ± SEM (*n* = 8/diet for each time point). a: Statistical difference between CT vs. HF, Et, and HFEt from the 10th day until the end of the protocol. b: Statistical difference between HFEt vs. HF and Et from the 17th day until the end of the protocol.

Control rats had a constant weight gain of 30 g per week during the first 3 weeks linked to normal growth, followed by a gain of 15 g per week until the end of the protocol. HF and Et rats showed similar weight gain curves (Figure 1A) but had a lower weight gain than the CT group. The difference became statistically significant at day 10 and remained until the end of the protocol, corresponding to a final difference of 19% for HF group and 16% for the Et group. HFEt rats exhibited the lowest weight gain relative to rats on other diets, leading to a final reduction of 28% compared to CT rats and a mean difference of 18% compared to HF and Et rats. The difference became statistically significant at day 17 compared to both HF and Et groups. It is important to notice that such significant differences in weight gain were observable while all diets were isocaloric and caloric intake tends to be equivalent between groups (Figure 1B).

### Plasma measurements

**Figure 2:**
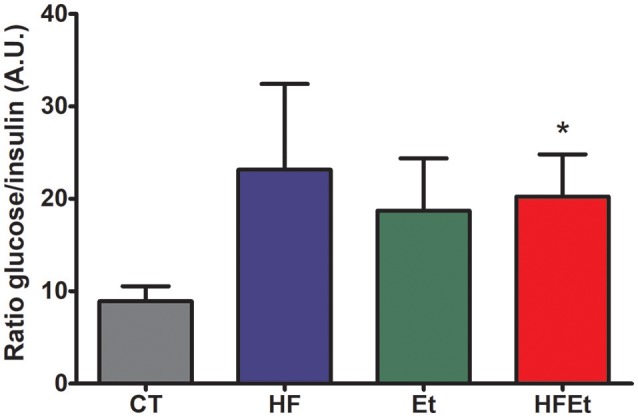
Ratio glycemia/insulinemia in rats after 6-week diet with either control (CT), high fructose (HF), ethanol (Et), or a mix diet (HFEt) and prior infusion of [1-^13^C]D-glucose. Glucose and insulin content were measured in a blood sample collected just before [1-^13^C]glucose infusion and after 6 weeks of diet to detect any disturbance in insulin and therefore glucose metabolism homeostasis at this time point. Values are mean ± SEM. ^*^Statistical difference between CT and HFEt.

Prior to the infusion of [1-^13^C]glucose, systemic glucose metabolism impairment was assessed in each group by determining the glycemia/insulinemia blood ratio. For the HF and Et groups, the ratio tended to be higher than in the CT group, while this difference was significant only for the HFEt rats (Figure 2).

Biochemical assays were also performed on plasma collected after the [1-^13^C]glucose infusion and are summarized in Table 2. No significant difference was observed between groups regarding proteins and albumin, which are nutritional markers indicating adequate dietary intakes. Total cholesterol, HDL-cholesterol and FFA were unchanged among groups. In contrast, plasmatic triglycerides (TG) tended to increase in the HF group and were significantly raised by 69% in the HFEt group.

**Table 2:** Biochemical assays on plasma in rats fed during 6 weeks with Control (CT), High Fructose (HF), Ethanol (Et), or a mix (HFEt) diet.

	**CT**	**HF**	**Et**	**HFEt**
Proteins (g/L)	57.0 ± 0.7	56.0 ± 2.8	52.0 ± 0.6	55.0 ± 0.8
Albumin (g/L)	27.0 ± 0.5	30.0 ± 0.8	26.0 ± 0.4	28.1 ± 0.6
Total Cholesterol (mmol/L)	1.84 ± 0.18	1.38 ± 0.13	1.35 ± 0.04	1.40 ± 0.12
HDL-Cholesterol (mmol/L)	0.79 ± 0.09	0.64 ± 0.12	0.65 ± 0.02	0.63 ± 0.06
TG (mmol/L)	1.33 ± 0.15	1.99 ± 0.63	1.05 ± 0.13	2.25 ± 0.31^*^
FFA (mmol/L)	0.92 ± 0.38	0.68 ± 0.27	0.80 ± 0.22	0.81 ± 0.13

* *Statistical difference between CT and HFEt*.

### Brain metabolism by HRMAS NMR spectroscopy

#### The specific enrichment of glucose (% ^13^C) in brain

**Figure 3:**
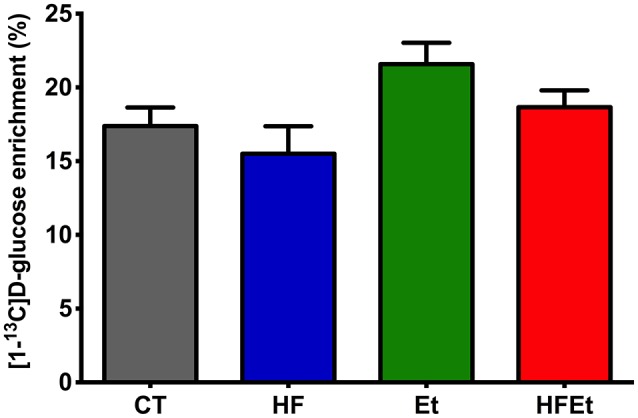
Specific enrichment of glucose ^13^C (% ^13^C) obtained in the brain following 1 h of [1-^13^C]D-glucose infusion in rats fed with control (CT), high fructose (HF), ethanol (Et), or a mix diet (HFEt). Specific enrichments were normalized to the Glucose C1 specific enrichment in each group.

The specific enrichment of glucose C1 (% ^13^C; Glc C1 SEnr) was determined on ^1^H spectra to evaluate the ^13^C-enrichment obtained in the brain following 1h of [1-^13^C]glucose infusion. Glc C1 SEnr in Et and HFEt groups were 21.6 and 18.7%, respectively, slightly higher than the one measured in CT (17.4%) and HF (15.5%) groups (Figure 3).

#### Relative enrichment of Glu C2/C3 and Gln C2/C3

**Figure 4:**
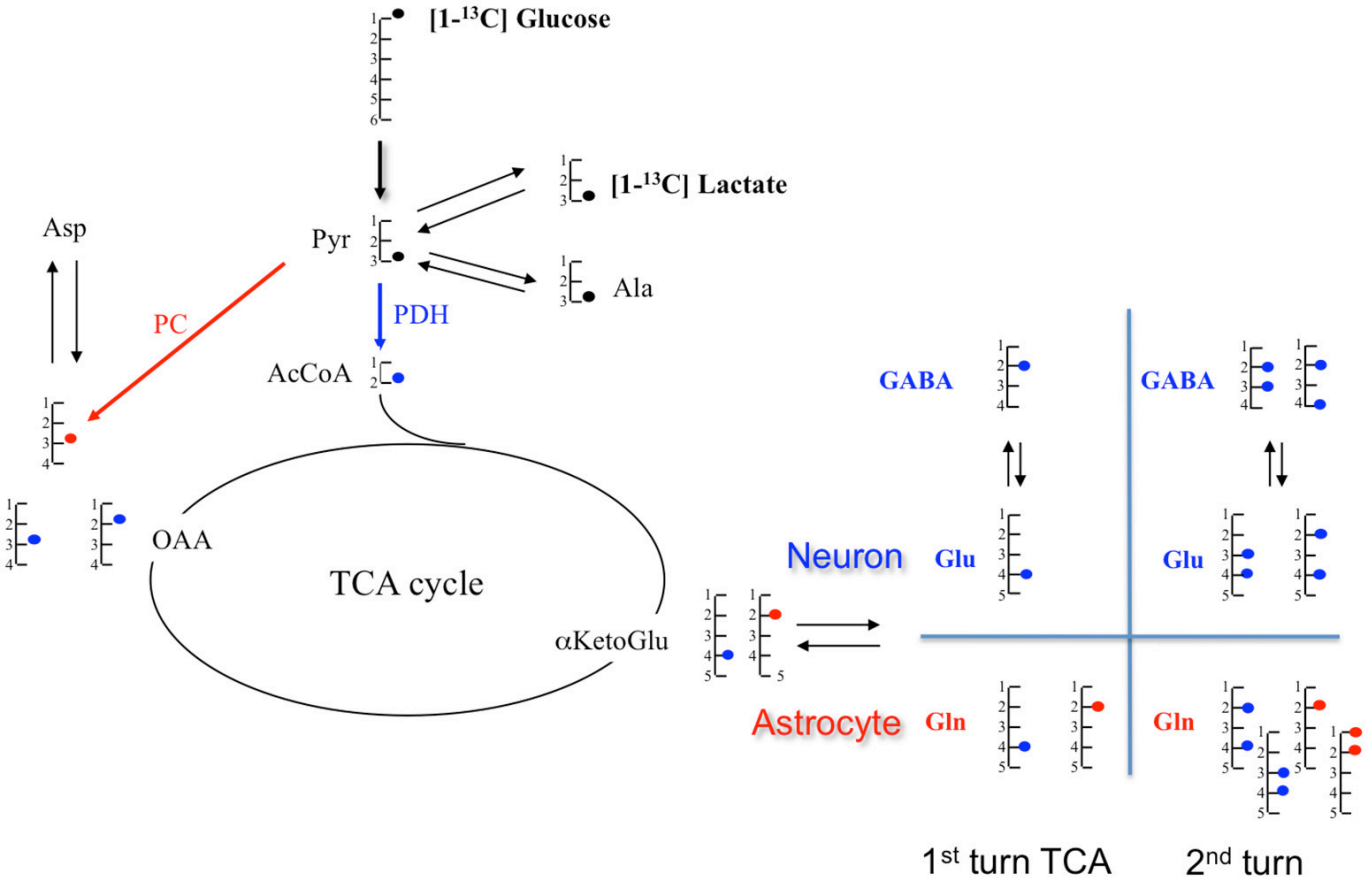
Schematic representation of metabolite labeling from [1-^13^C]glucose during the first tricarboxylic acid (TCA) cycle turn. In astrocytes, [1-^13^C]glucose follows either the pyruvate dehydrogenase (PDH; 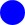) or the pyruvate carboxylase (PC; 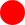), but only the PDH pathway occurs in neurons. At the end of the glycolysis, [1-^13^C]glucose provides [3-^13^C]pyruvate which can take two paths: either pyruvate dehydrogenase (PDH; 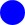) or pyruvate carboxylase (PC; 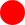). In the PDH pathway, [3-^13^C]pyruvate gives [2-^13^C]AcCoA which enters in the TCA cycle and leads to [4-^13^C]citrate, then to [4-^13^C] αKG. This intermediate αKG forms [4-^13^C]glutamate, which is metabolized in [4-^13^C]glutamine. Through TCA cycle, by the symmetry of some intermediates, [4-^13^C]αKG gives two isotopomers of OAA: [2-^13^C]OAA (50%) and [3-^13^C]OAA (50%). Then, it provides Asparate labeled in [2-^13^C] (50%) and [3-^13^C](50%). In the PC pathway (only in astrocyte compartment), [3-^13^C]pyruvate is converted in [3-^13^C]OAA which enters TCA cycle to form [2-^13^C]αKetoGlu which leads to [2-^13^C]glutamate and then to [2-^13^C]glutamine. α-KG, α-ketoglutarate; Ala, alanine; AcCoA, acetyl-CoA; Glu, glutamate; Gln, glutamine; GABA, γ-aminobutyric acid; Lac, lactate; OAA, oxaloacetate.

From ^13^C spectra, the relative enrichments (comparison of ^13^C incorporation between different carbons in the same molecule) for glutamate and glutamine were determined. Classically, glutamate, present in greater quantity in neurons, represents the neuronal compartment whereas glutamine, synthetized only in astrocytes, represents the astrocytic compartment. Therefore, these measurements are used to determine if [1-^13^C]glucose is preferentially metabolized in astrocytes (C2/C3 Gln>1; due to the activity of pyruvate carboxylase (PC) present only in astrocytes) or in the neuronal compartment (C2/C3 Glu around 1; no pyruvate carboxylase activity) (For a schematic representation of the fate of ^13^C see Figure 4 in which labeling linked to PC is marked as an open circle whereas the one linked to pyruvate dehydrogenase is represented by a filled circle). No variation in the Glu C2/C3 ratio was observed between groups (Table 3). Compared to the CT group, the Gln C2/C3 ratio was significantly increased by 30.6% in HF, 36.1% in Et and 28.7% in HFEt groups, which indicates an increase in the astrocytic PC activity.

**Table 3:** Ratios C2/C3 glutamate and C2/C3 glutamine measured by ^13^C NMR HRMAS in brain perchloric extract of rat who followed Control (CT), High Fructose 20% (HF), Ethanol (Et), or a mix diet (HFEt).

	**CT**	**HF**	**Et**	**HFEt**
C2/C3 glutamate	1.15 ± 0.12	1.11 ± 0.05	1.15 ± 0.05	1.20 ± 0.10
C2/C3 glutamine	1.08 ± 0.08	1.41 ± 0.09^*^	1.47 ± 0.11 ^*^	1.39 ± 0.08 ^*^

* *Statistical difference between CT and HF and/or Et*.

#### ^13^C enrichment of brain metabolites

**Figure 5:**
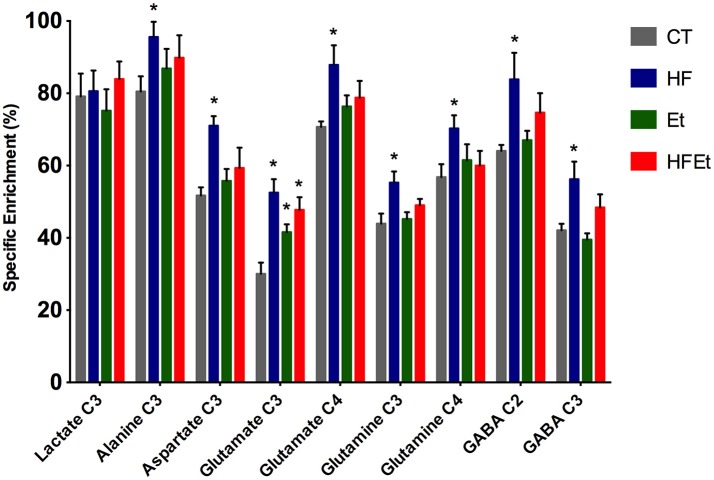
^13^C-specific enrichments (%) of some carbon positions of some brain metabolites after 1 h infusion of [1-^13^C]glucose in rats fed with control (CT), high fructose (HF), ethanol (Et) or mixed (HFEt) diet. ^*^Statistical difference between CT and HF.

From POCE spectra, brain metabolite ^13^C enrichments (percentage of ^13^C incorporated in a metabolite at a specific carbon position) were measured (Figure 5). In each diet group, Glc C1 SEnr previously measured was used to normalize ^13^C enrichments of brain metabolites and thus compare groups between them. Most of the cerebral metabolites ^13^C enrichments increased significantly in the HF group compared to the CT group; alanine C3 +19% (*p* = 0.03), aspartate C3 +37% (*p* = 0.002), glutamate C3 +75% (*p* = 0.005), glutamate C4 +24% (*p* = 0.05), glutamine C3 +26% (*p* = 0.03), glutamine C4 +24% (*p* = 0.05), GABA C2 +31% (*p* = 0.04), GABA C3 +34% (*p* = 0.02). In comparison to the CT group, brain metabolites ^13^C enrichments in the Et group tended to increase, except for lactate and GABA. Concerning the mix diet HFEt, it caused an increase of a magnitude, which is intermediate between the effect observed for the HF and Et groups for all measured amino acid carbons, except for glutamine C4.

## Discussion

In the present study, we have evaluated the effects of an intake of fructose (similar as in the Western diet) and/or of a moderate amount of ethanol on cerebral glucose metabolism in young adult rats. Indeed, the brain represents only 2% of the body mass but it consumes up to 25% of the circulating glucose at rest as its main energy source (33). Rodents are a good model to study the effects of alcopop consumption in young people, as they exhibit a very similar fructose metabolism than humans (34, 35). Moreover, the time window for “adolescence” (from 30 to 46 postnatal day) (36) and “late-adolescence” (from 46 to 59 postnatal day) are very convenient and can be related to their human counterparts (23). With our experimental design, we studied the effects on metabolic pathways of fructose and ethanol substrates *per se* in the case of a normal and isocaloric diet in rats from late-adolescence (postnatal day 53) to adulthood (13 weeks). The caloric intake from beverages in the HFEt group was equivalent to human alcopop consumption of 300 ml per week containing on average 4% of alcohol (185 kcal/week/rat).

### Effect of the diets on body weight

Compared to the control group, we measured a lesser weight gain in all treated groups (HF, Et, and HFEt). Since rats were both isocaloric and *pair-fed* (same amount of caloric intake for all rats independently of their body weight), our results strongly suggest that fructose has a lower energetic efficiency than control chow diet. This weaker weight gain observed in HF fed rats seems at variance with reports of either similar body weight (37–39) or even increased body weight reported in the literature [review in (40)]. However, these differences with others can be explained by our experimental design, which differed from previous studies that used up to 60% fructose in the diet (20% fructose here) and/or the diet was available *ad libitum*. Nonetheless, in one study performed by Alwahsh et al. (30), conducted in adult Sprague-Dawley rats, a decrease in body weight was measured after 4 and 8 weeks of high fat and 70%-fructose diet. More interestingly, in this study, liver weights did not change between control and rats that underwent the diet, whereas the ratio liver weight/body weight was increased, indicating a change in body composition. Two other studies have shown that fructose diet didn't lead to increased weight when directly administered during 4 months to adult mice [15% fructose in the diet; (41)] or in rat pups after maternal supplementation (42).

In our study, as a consequence of its design and the choice to highlight the substrate effect, no effect linked to an excessive caloric intake can be evidenced. However, after a 6-week period on a 20%-fructose diet, we observed an increase in visceral adipose tissue (data not shown). Taking all together (lesser body weight gain and increase in visceral adipose tissue), our data suggest either a decrease in rat growth and/or a modification of the body composition, a consequence of a decrease in energy efficiency linked to fructose and/or ethanol intake. Since it has been shown that excessive consumption of fructose increases reactive oxygen species (ROS) production and mitochondria impairment in skeletal muscle [for review, see (43)], which could therefore lead to tissue damage, we may hypothesize that change in body composition could be a consequence of such ROS production and either less muscle weight gain or a decrease in global energy efficiency. Moreover, aged rats having a 30% fructose supply during 5 months evidenced an accelerated loss of muscle mass vs. control group by altering the stimulation of postprandial protein synthesis (44); this effect perhaps due to a decrease in insulin sensitivity could participate to a lesser growth in young rats needing a positive energetic balance. A short period of 1 week of high fructose is sufficient in humans to alter the expression of genes involved in the energetic metabolism in skeletal muscle (45).

Concerning the Et diet, we observed a lower body weight gain, a result already found in a previous study in which a lower weight was measured at postnatal day 114 in rats exposed chronically to ethanol vapor since adolescence (22). Such a variation in body weight was also measured after a 20-days exposure to 10%-ethanol gelatin (46).

In the HFEt group, the decreased body weight gain was even more extensive, indicating a synergistic effect of both diets, which is consistent with a previous study showing a combined fructose-ethanol diet resulted in a more apparent decreased body weight gain during the 2nd and 3rd weeks of feeding compared to ethanol group (30).

### Effect of the diets on plasma lipid contents, glycemia, and insulinemia

We did not observe any statistical difference in plasmatic markers of lipid metabolism (total cholesterol, HDL-cholesterol, and FFA) between CT, HF and Et groups. Nevertheless, we measured an increase in triglyceridemia in the HFEt group (and a tendency to increase in the HF group), which is in accordance with the fact that high fructose diet can induce dyslipidemia (11, 14, 16) and early liver fatty acid deposit with lipid microvacuoles, a phenomenon that we observed after 6 weeks of diet (Supplementary Figure 1).

A higher dose of fructose and ethanol (30% of each substrates in the diet for 4 weeks) has been shown to significantly elevate blood insulin (31). In our study, plasmatic ratio glucose/insulin was increased after all diets, compared to control, but this increase was only significant in the HFEt group. This increase indicates that insulin homeostasis has been disturbed at the examined time point. It's now well-known that fructose and ethanol diets alter hepatic metabolism (47, 48). By contrast to glucose, fructose and ethanol do not require insulin for their metabolism (29) and it has been shown that both molecules can impair insulin sensitivity (49–53), a result observed in our study.

### Effect of fructose on brain oxidative metabolism

Fructose enters the brain through Glucose Transporter 5 (GLUT5), which is found in the blood-brain barrier endothelial cells (54). In the brain, GLUT5 is expressed in microglial cells (55–57). However, some studies have found that GLUT5 can also be expressed in Purkinje cells in mouse cerebellum, together with the specialized enzymes ketohexokinase (fructokinase), aldolase, and triokinase, three enzymes important for the fructose-specific metabolic pathway (58). In a more recent study conducted in rats, in addition to microglia and Purkinje cells, GLUT5 was localized in hypothalamic neurons as well as in tanycyte processes (57). However, even if fructose was shown to be metabolized by the brain (59), its metabolism is low compared to the one of glucose.

^13^C-NMR spectroscopy is a powerful tool to study metabolism but with a low sensitivity. Only molecules above the mM range can be detected and it also requires the infusion of ^13^C-labeled precursor. In this study, [1-^13^C]glucose infusion allowed the following of brain glucose metabolism [for an overview of the fate of ^13^C in brain metabolism, see (60, 61)], the main brain substrate, and was chosen to reflect the impact of the diet. Indeed, if any metabolic adaptation was established during fructose and/or ethanol intake, we will be able to observed it through the [1-^13^C]glucose pathway and ^13^C distribution. For the first time, a significant raise in ^13^C-specific enrichment in intermediate metabolites (alanine, aspartate, glutamate, glutamine, and GABA) has been measured in HF rats, as well a small raise in Et group and an intermediate increased enrichment for HFEt animals. However, this increase was only statistically significant in the HF group. This means that even if ethanol and ethanol+fructose may modify metabolism, the major impact was linked to the fructose diet.

^13^C-labeling of glutamate and aspartate reflects neuronal glucose oxidative metabolism through the TCA cycle with formation of ^13^C-labeled α-ketoglutarate and oxaloacetate, from which these two amino acids are formed, respectively. Since glutamine synthetase is present only in astrocytes (62), labeling of glutamine reflects astrocytic oxidative metabolism. An increase in the specific enrichments of these amino acids indicates that oxidative metabolism was increased in the brain after fructose dietary supplementation. Interestingly, lactate C3 specific enrichment was unchanged whereas alanine C3 specific enrichment was increased. Alanine enrichment reflects the one of pyruvate. This indicates that pyruvate, formed at the end of the glycolysis, is mainly directed toward oxidative metabolism rather than lactate production, a pathway that is predominant in astrocytes (63). Finally, glutamate and glutamine C3 specific enrichments also increased. Since these carbons are labeled during the second TCA cycle turn, an increase of ^13^C incorporation into these carbon positions indicates that the TCA cycle is turning faster (C3/C4 ratios increased). Taken altogether, our data show a relative increase in oxidative metabolism compared to glycolytic lactate production after the fructose diet.

Such an increase in mitochondrial metabolism was already observed when insulin-resistance is present (64–66). Another study, in which fructose was administered during gestation and lactation, (42) demonstrated that maternal exposure to fructose led to a significant increase in state 3 respiration in rat pup brains, as well as a decrease in the Phosphate/Oxygen (P/O_2_) ratio, indicating a lower efficiency of the respiratory chain and an increase in mitochondrial metabolism to compensate for this lower ATP production (mitochondrial decoupling).

### Effect of fructose on astrocytic metabolism

In the brain, it has been proposed that a metabolic compartmentation occurs, the astrocytes being more glycolytic and the neurons more oxidative. This leads to a lactate shuttle between astrocytes and neurons, a metabolic cooperation still debated nowadays but more and more admitted [for a review see (67). In our study, we found that lactate C3 specific enrichment was unchanged whereas specific enrichments of amino acids linked to the TCA cycle were increased. This relative increase in oxidative metabolism compared to glycolytic lactate production after the fructose diet may have an impact on cognitive functions. Indeed, it has been shown that fructose diet may impact memory (41), and, in parallel, that lactate shuttling between astrocytes and neurons through the monocarboxylate transporters (MCT) is necessary for long-term memory formation (68). In a more recent study, the down regulation of the neuronal lactate transporter, MCT2, in the barrel cortex of rat suppressed the blood-oxygen-level dependent (BOLD) effect observed in functional magnetic resonance imaging (MRI) when the whisker were activated, suggesting impairment of the synaptic activity when this lactate shuttle is suppressed (69).

The metabolic modification after the HF diet can also be observed through the relative enrichments of the different glutamine carbons. Indeed, glutamine C2/C3 ratio increased in the HF group. An increase in this ratio reflects an increase in the pyruvate carboxylase (PC) pathway (70). PC is the predominant anaplerotic enzyme in the brain and is exclusively located in astrocytes (71). This enzyme allows the conversion of pyruvate directly into oxaloacetate and is activated by AcCoA. Therefore, an increase in the glutamine C2/C3 ratio indicates that a greater amount of pyruvate is directed toward the TCA cycle rather than being converted into lactate. These data strengthened the conclusion that fructose diet alters metabolism in astrocytes. The relative decrease in lactate production compared to oxidative metabolism after fructose diet suggests a remodeling of astrocytic metabolism, a decrease of glycolysis in these cells, which may have a role in this memory impairment. This hypothesis is supported by preliminary results obtained by *in vivo* diffusion MRI on HF rat brains after 5 weeks of diet: a 18, 13, and 20%-decrease in fractional anisotropy were measured (Supplementary Figure 2) compared to control in the cortex, hippocampus and striatum, respectively, indicating a decrease in fiber organization and/or myelination (72) in a period of critical brain maturation.

## Conclusion

In this study, we followed the effects induced by a high fructose diet (20%) combined or not to a moderate amount of alcohol in young rats. We provide here evidence that in the case of a combined fructose and moderate dose of ethanol consumption, under isocaloric conditions, fructose has the biggest impact on brain metabolism and increases oxidative metabolism. These early alterations show that the brain is vulnerable at the metabolic level to fructose consumption during late-adolescence throughout adulthood in rats. Brain metabolic remodeling appears to occur precociously without a concurrent increase in overall energy intake and seems consequently to be linked to fructose itself. These disturbances should be more explored to elucidate deleterious long-term outcomes, such as memory impairments, as recently proposed (73).

## Author contributions

DE and A-KB-S: Performed experiments; DE, J-LG, HG, M-CB and A-KB-S: Performed interpretation and wrote article.

### Conflict of interest statement

The authors declare that the research was conducted in the absence of any commercial or financial relationships that could be construed as a potential conflict of interest.

## Supplementary material

Supplementary Figure 1 Histology of liver from control **(A)** and HF rats **(B)** fed with control diet or 20%-high fructose diet during 6 weeks. Representative hematoxylin–eosin–saffron (HES) and Periodic Acid Schiff (PAS) stainings. Inclusion of lipid droplets are detected in **(C)**.

Supplementary Figure 2 Fractional anisotropy values measured from diffusion weighted MRI in the cortex, striatum and hippocampus of control rats (CTL) and rats fed with a high fructose diet during 5 weeks (HF). ^*^Statistical difference between CTL and HF.
