# Supplementary figures and images for: Consumption of Alcopops During Brain Maturation Period: Higher Impact of Fructose Than Ethanol on Brain Metabolism
Source: Front Nutr. 2018 May 8;5:33. doi: 10.3389/fnut.2018.00033 (PMC5952002; doi:10.3389/fnut.2018.00033)

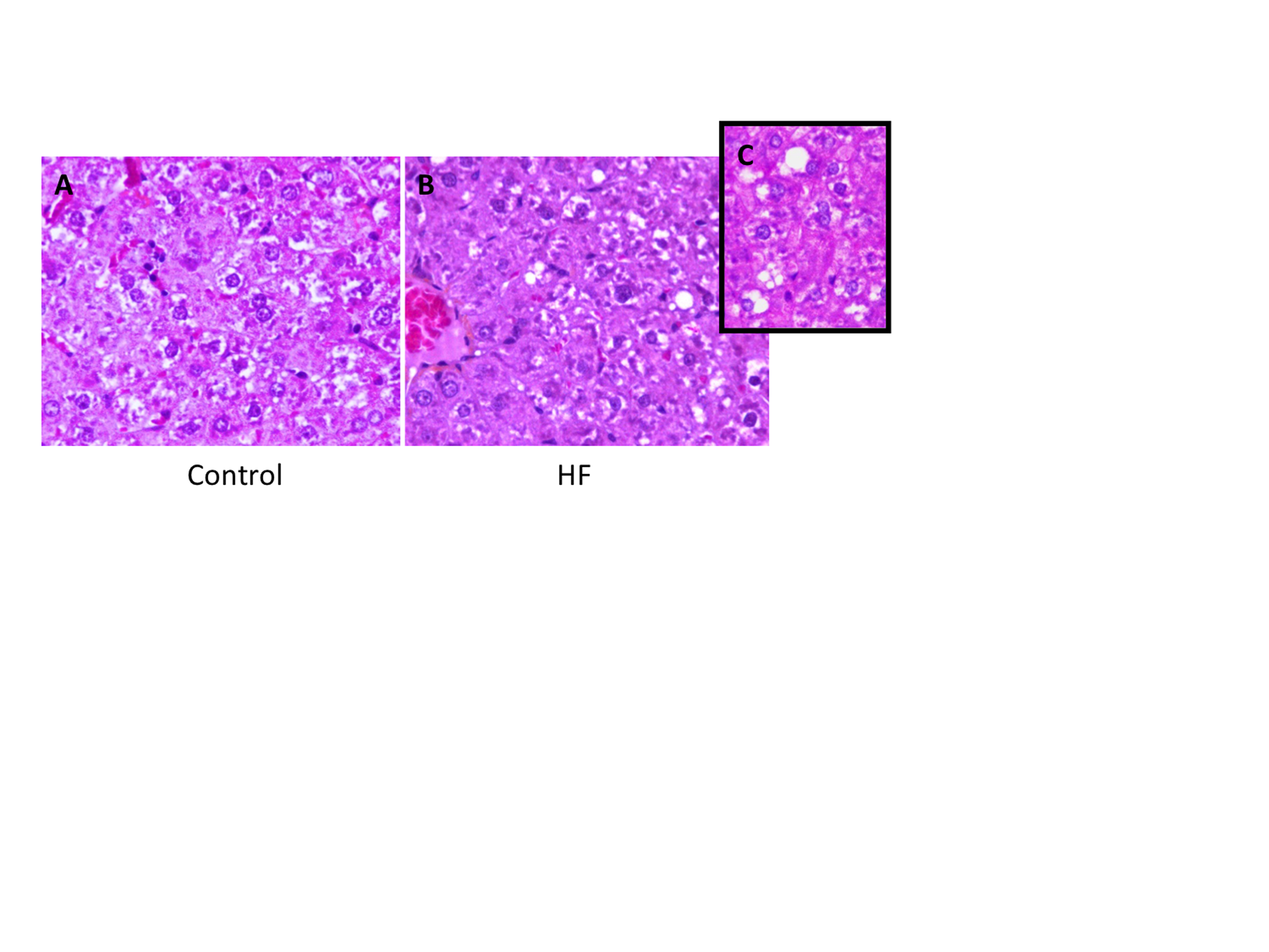

Supplement: Supplementary Figure 1 — Histology of liver from control (A) and HF rats (B) fed with control diet or 20%-high fructose diet during 6 weeks. Representative hematoxylin–eosin–saffron (HES) and Periodic Acid Schiff (PAS) stainings. Inclusion of lipid droplets are detected in (C). [file Image1.TIFF]

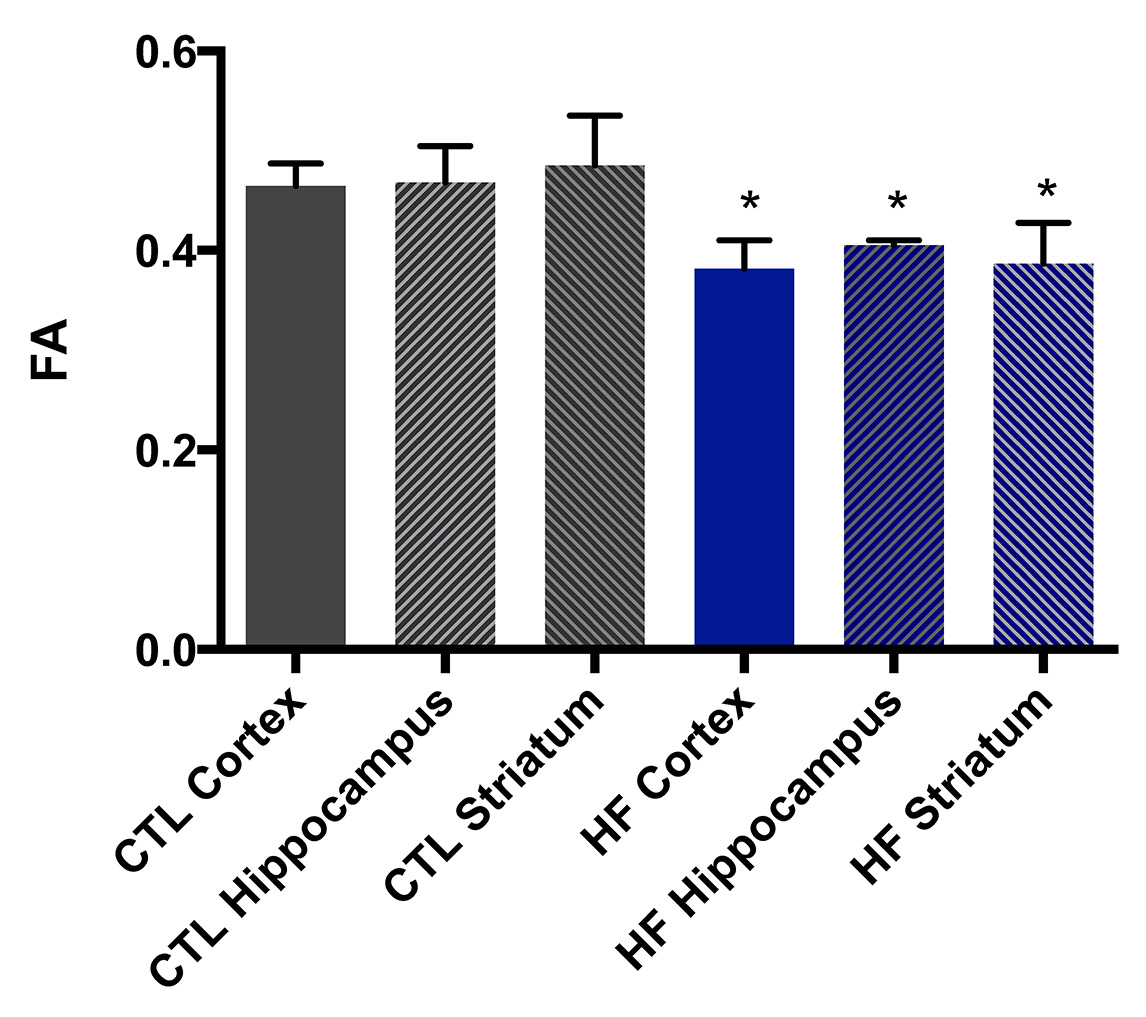

Supplement: Supplementary Figure 2 — Fractional anisotropy values measured from diffusion weighted MRI in the cortex, striatum and hippocampus of control rats (CTL) and rats fed with a high fructose diet during 5 weeks (HF). *Statistical difference between CTL and HF. [file Image2.TIFF]
